# U-Net-Based Models towards Optimal MR Brain Image Segmentation

**DOI:** 10.3390/diagnostics13091624

**Published:** 2023-05-04

**Authors:** Rammah Yousef, Shakir Khan, Gaurav Gupta, Tamanna Siddiqui, Bader M. Albahlal, Saad Abdullah Alajlan, Mohd Anul Haq

**Affiliations:** 1 Yogananda School of AI, Computers and Data Sciences, Shoolini University, Solan 173229, India; rammah.y7y@gmail.com; 2 College of Computer and Information Sciences, Imam Mohammad Ibn Saud Islamic University (IMSIU), Riyadh 11432, Saudi Arabia; bmalbahlal@imamu.edu.sa (B.M.A.); saalajlan@imamu.edu.sa (S.A.A.); 3 Department of Computer Science and Engineering, University Centre for Research and Development, Chandigarh University, Mohali 140413, India; 4 Department of Computer Science, Aligarh Muslim University, Aligarh 202001, India; tsiddiqui.cs@amu.ac.in; 5 Department of Computer Science, College of Computer and Information Sciences, Majmaah University, Al-Majmaah 11952, Saudi Arabia

**Keywords:** U-Net, MR brain images, loss functions, image segmentation, optimization, deep learning, machine learning

## Abstract

Brain tumor segmentation from MRIs has always been a challenging task for radiologists, therefore, an automatic and generalized system to address this task is needed. Among all other deep learning techniques used in medical imaging, U-Net-based variants are the most used models found in the literature to segment medical images with respect to different modalities. Therefore, the goal of this paper is to examine the numerous advancements and innovations in the U-Net architecture, as well as recent trends, with the aim of highlighting the ongoing potential of U-Net being used to better the performance of brain tumor segmentation. Furthermore, we provide a quantitative comparison of different U-Net architectures to highlight the performance and the evolution of this network from an optimization perspective. In addition to that, we have experimented with four U-Net architectures (3D U-Net, Attention U-Net, R2 Attention U-Net, and modified 3D U-Net) on the BraTS 2020 dataset for brain tumor segmentation to provide a better overview of this architecture’s performance in terms of Dice score and Hausdorff distance 95%. Finally, we analyze the limitations and challenges of medical image analysis to provide a critical discussion about the importance of developing new architectures in terms of optimization.

## 1. Introduction

Deep learning has become of significant interest and utilization for medical image analysis in recent years by virtue of advancements in computer vision. Despite this growth, deep learning in medical imaging still faces challenges that need to be addressed and diminished.

Image segmentation, in general, means isolating and portioning the image into different classes (organ, tissues, biological structure, etc.) into meaningful segments, and it involves both classification and detection, where, in segmentation, we localize and classify a group of pixels corresponding to a specific class. Most classification methods depend on intensities, merely information, or a combination of objects’ higher structural information and their intensities. Medical image segmentation consists of anatomical structure labeling within templates, then image registration algorithms are used to map the templates to a particular subject domain through non-linear transformations. After that, a voting process (or maybe a more sophisticated method such as Bayesian weighting) is applied to select the correct label from the labels space and assign it to the segments. The new scheme of deep learning as a main category of artificial intelligence is used for a variety of medical applications such as monitoring neurological disorder patients [1], pneumonia classification using chest X-ray [2], brain tumor classification [3], breast cancer [4,5,6], COVID-19 detection from chest CT scans [7,8], and other non-medical applications [9] such as hate speech prediction [10,11,12] The deep learning approach for medical imaging segmentation includes the multi-level features representation from image intensities. The ability to learn features representation through non-linear transformation is a plus point for deep learning compared to machine learning algorithms, where there is less dependency on the prior knowledge of the field of application. MRIs are used as a major method of diagnosis for different diseases such as breast cancer [13,14] and prostate cancer [15,16]. Eventually, deep learning applications based on CNNs have become the dominant methods for biomedical image recognition and applications [17].

A deep learning limitation in the medical image application is the computational complexity due to the large data sizes and the large variance. Therefore, many methods were proposed towards this issue, for example, Prasoon A et al. [18] have proposed a tri-planar concept to mitigate the complexity of 3D CNNs. However, image pre-processing has taken on a significant role in reducing the computational power needed. Another issue related to the segmentation task of medical images, especially 3D MRI, is that it is a time-consuming process, and it is subtle for faults due to the interoperable variations. Automated delineation will provide a faster and reliable systematic way of obtaining desired objects from MRI images. Siddique N et al. [19] have provided a comprehensive review of different U-Net models used for different modalities of medical image segmentation. U-Net has demonstrated the transformation of the entire concept of segmentation by elevating the accuracy, which made it the mainstream method used recently in research towards optimal results.

The contributions of this paper are summarized as:

1. Addressing the recent techniques focused on brain tumor segmentation based on U-Net architecture as the backbone, along with its variants.
2. Highlighting the major trends and patterns in the research that may help to guide future work in the field by summarizing the cutting-edge techniques in one place.
3. Providing a comparative analysis of the most recent relevant literature results and other experimental results to observe the improvements achieved by the incremental research.

The paper is organized as follows. First, we briefly explain the concept of brain tumor segmentation from MRIs. Secondly, we discuss in detail the main U-Net-based architectures. Thirdly, we demonstrate network performance aspects such as loss functions and evaluation metrics, which are used for such application of deep learning, and we also provide a comparative analysis of the U-Net variants for evaluation purposes, in addition to the experimental results listed using four U-Net models. Finally, the discussion and conclusion, including the limitations and challenges, are summarized in Section 4 and Section 5, respectively.

### 1.1. Brain MRI Segmentation

Magnetic resonance images (MRI) is a medical technique that uses magnetic field radio waves to generate images that contain details more than normal images, and it is specified for biological organs and tissues in the human body. Lu SY et al. [20] have proposed a model based on transfer learning, which detects abnormal brain growth.

Since this imaging technique contains high-resolution detailed images in a 3D shape, these images are a non-invasive way to analyze and examine body organs, tissues, and skeletal structures, and this, in turn, helps doctors to diagnose a variety of problems and diseases. Brain MRI, in particular, is used to diagnose common problems such as tumors, strokes, multiple sclerosis, eye and ear disorders, aneurysms of cerebral vessels, and other brain injuries.

MR image acquisition, in general, requires standardization to avoid the phenomenon of “distribution drift” due to the variety of scanning tools and mechanisms. In a traditional analysis of brain neuro MRI [21], a radiologist studies the image and generates his report for the doctor of his views. Medical images are significant for further treatment also, with respect to different treatment methods such as surgeries, chemotherapies, and radiotherapies [22,23].

**Figure 1:**
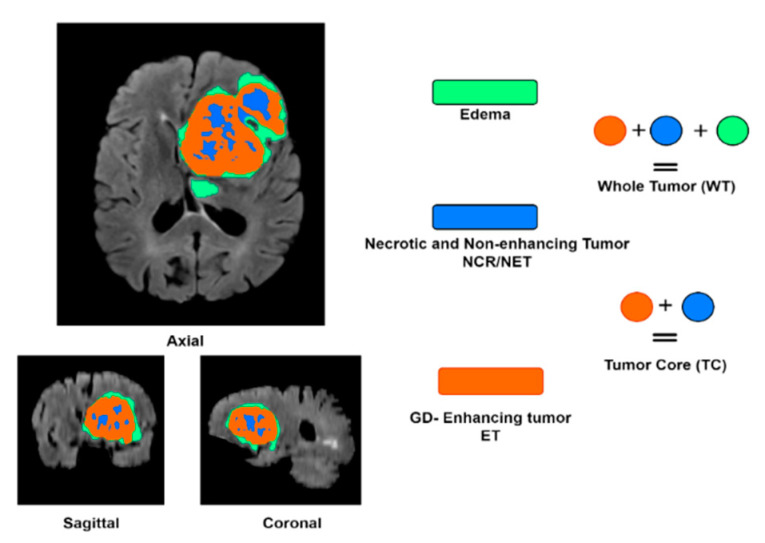
MRI brain tumor modalities and pathophysiology subregion labels.

A brain tumor MRI example has three main regions, named whole tumor, which represents the total tumor size, including the edema, the enhanced tumor, and the necrotic and non-enhanced tumor (WT = ET + ED + NCR/NET); tumor core (TC = ET + NCR/NET); and enhancing tumor (ET = ET), as shown in Figure 1.

### 1.2. Before U-Net

The general concept of segmentation tasks before U-Net used the “sliding window” method for prediction of each pixel’s class label when this pixel is the center of the sliding window (patch). However, this method was considered inefficient due to the time consumed for the sliding window to scan the whole image and the redundancy caused by overlapping between patches. Another weakness in this method was the patch size tuning to accomplish the tradeoff between the spatial localization accuracy and the context usage [24]. The major key differences between the U-Net models and the traditional models involve the followings points:

- Architecture: traditional deep learning models, such as fully convolutional networks (FCNs) or convolutional neural networks (CNNs), typically have a simpler architecture compared to U-Net-based models.
- Training Data: U-Net-based models are specifically designed to work well with medical imaging data, which often have higher resolutions and more complex structures than natural images. Meanwhile, traditional deep learning models may struggle to handle complex data and may need to be fine-tuned to work well with medical imaging data.
- Performance: U-Net-based models have been shown to perform better than traditional deep learning models on brain tumor segmentation tasks, particularly on datasets with limited training data.
- Small objects segmentation: U-Net-based models have the capacity to handle small structural objects in the image, which is an important aspect in brain tumor segmentation where small tumors need to be segmented.

In summary, U-Net-based models have been particularly designed to work well with medical images, and they have demonstrated superior performance in brain tumor segmentation tasks compared to traditional deep learning models.

## 2. U-Net and U-Net Expansions towards Optimized DL Models for Segmentations

### 2.1. U-Net

After the brief introduction about the basic structure of deep networks and CNNs, it will be easier to understand U-Net models. U-Net is a lead model for image segmentation developed by Olaf Ronnenberg et al. [25] in 2015. It was first introduced for biomedical image segmentation after many segmentation attempts have failed to segment medical images. U-Net attained its popularity because of its accurate results and performance and because it requires a smaller amount of training data, which is considered the major issue for medical images.

The basic structure of this model consists of two main paths, and it is most similar to the auto-encoder architecture, where the left path (encoder) is called the contracting or compressive path, and its structure is based on a regular CNN deep network. The second path is the decoder or the expanding path (up-sampling or synthesis path in some references), and this part of the network consists of both deconvolutional and convolutional layers. Since the contracting path down-samples the input images, the expanding path recovers the input image resolution and spatial structure using some optimized techniques such as concatenating skip connections. In the expansion path, the network learns spatial classification information by generating dense predictions in a higher resolution. In addition to that, it increases the resolution of the output, which further is passed to the final convolutional layer for creating the segmented image in the same shape as the input image. In other words, the network processes the image with shape (h, w, n) to generate an output image (h, w, n) but with the segmented region highlighted (the area of interest, e.g., brain tumor), which means preserving the same input shape.

In medical image analysis, the classification task [26] is important, but it does not provide the pixel-level context representation, which is more important because classification will only classify the entire image into one label.

U-Net and the further optimization techniques deal with multi-dimensional tensors (three or four mostly) as inputs, and the final output of the network will preserve the same input shape. Since U-Net has been proposed, it has been the base of the majority of research for medical image segmentation, and many advancements have been developed either by modifying the base architecture or by incorporating other architectures into U-Net.

#### U-Net Workflow

- *The Contracting Path*

**Figure 2:**
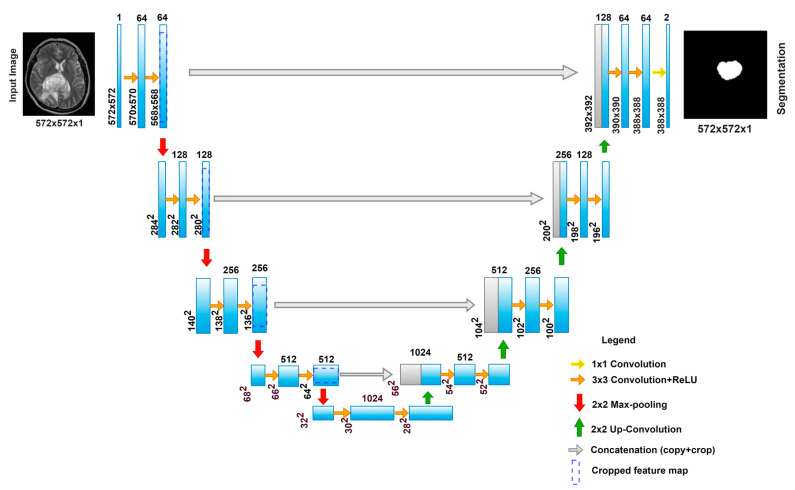
Basic U-Net structure for brain tumor segmentation.

As mentioned earlier, the contracting path follows a typical CNN network, which consists of two (3 × 3) successive convolutions followed by non-linear activations (e.g., ReLU) and then by a max pooling layer. This same structure is repeated furthermore times until reaching the bottleneck. In the contracting path, dimensions are reduced because of the strided convolutions and pooling layers, but the channel number and the receptive field are increased, as shown in Figure 2.

- *The Expansion Path*

The novelty of the U-Net comes from this path, where up-sampling of feature maps from the bottleneck consists of (2 × 2) up-convolutions to recover the dimensions of the input image. Each stage of the expansion path contains (2 × 2) up-convolution and normal (3 × 3) convolutional and ReLU activations. At each up-sampling in this path, the number of channels is reduced to half, while the up-convolution increases the width and height of the image.

To increase the dimensions of the image after each (2 × 2) up-convolution, a concatenation from the same level layer in the contracting path of the feature map is added after cropping, though the spatial features are recovered. The cropping before concatenating the feature map between the paths is necessary because the pixel features at the borders have less contextual information. Repeating these arrangements as the number of stages in the contracting path, taking into consideration the concatenating connections between the corresponding stages from both paths, we reach the last layer in this architecture. At the last layer of this architecture, there is a 1 × 1 convolution that reduces the feature map to match with the right number of channels and generates the final segmented image with the desired number of classes and the same resolution as the input resolution.

- *Training*

Stochastic gradient descent (SGD) is used for training the network, and to evaluate model in the last layer, the energy function is calculated using the SoftMax pixel-wise over the final feature map (after the 1 × 1 convolution layer) where the loss function used is the cross-entropy loss function. The SoftMax pixel-wise function is given by:

(1) $$p_{k}\left(x\right)=\frac{\exp\left(a_{k}\left(x\right)\right)}{\sum_{k^{\prime }=1}^{K}\exp\left(a_{k}{\left(x\right)}^{\prime }\right)}$$

where *a_k_*(*x*) is the function of activation corresponding to channel (*k*) and a pixel position at (*x*). *K* is the number of classes (labels within the segmented image).

However, the energy function, which is the cross entropy that penalizes at each spatial location, is defined as:

(2) $$E=\sum_{x\in }\omega \left(x\right)log(p_{\ell \left(x\right)}\left(x\right))$$

where (*Ɩ*) is the exact label of each pixel. *w*(*x*) is the weight map, which is defined as:

(3) $$\omega \left(x\right)=\omega_{c}\left(X\right)+\omega_{0}\cdot \exp\left(-\frac{{\left(d_{1}\left(X\right)+d_{2}\left(X\right)\right)}^{2}}{2\sigma^{2}}\right)$$

where *ω_c_* is the weight map used for class frequencies balancing. *d*_1_ is the distance between the nearest cell and the border, while *d*_2_ is the distance between the border and the second nearest cell.

### 2.2. 3D U-Net

One of the first optimization techniques used after U-Net was the 3D U-Net in 2016, and it was published as MICCAI 2016 for volumetric segmentation [27]. Similar to the original U-Net discussed above, 3D U-Net has the same structure, and it comprises the contracting (analysis) path and the expanding (synthesis) path. The main difference between both architectures is the use of 3D convolutional and pooling operations. For example, in the analysis path, each layer includes 3 × 3 × 3 convolutions followed by non-linear activations (ReLU) and a (2 × 2 × 2) max-pooling operation. On the other hand, the synthesis path consists of 2 × 2 × 2 up-convolutions and strides of two in each dimension followed by two successive 3 × 3 × 3 convolutions and then non-linear activations (ReLU). When using 3D segmentation, less annotated images are required due to the redundancy resulting from the repeating structures and shapes within the volume channels, therefore, faster training with scarcely annotated data is efficient. After 3D U-Net was proposed, the majority of research adopted it extensively with 3D volumetric CT scans and MR image segmentation for two main applications, with the first being diagnosing diseases such as cardiac structures [28], brain tumors [29,30,31], liver tumors [32,33], and bone structures [34]. Moreover, many other applications fall into the two preceding mentioned fields. Further optimized methods based on 3D U-Net have been used for more effective biomedical image segmentation. Zhuqing Yang [35] has introduced the self-excited compressed dilated convolution (SECDC) module based on the 3D U-Net network because there was the problem of complex structure, which leads to high calculation capacity required, and the new module helps by reconstructing high precision lightweight segmentation models. As a result, therapy calculations are reduced and their results on the BraTS 2019 dataset have achieved state-of-the-art results by using less data.

### 2.3. Residual U-Net

This architecture comes from the combination of the Residual-Net [36] and the basic U-Net. Rolling back to the original Res-Net, the main idea was to train deeper networks because adding layers increases the complexity and the computational power, plus it causes the vanishing gradients problem too. A residual network was used for brain cancer classification from MRI [37].

Residual Blocks:

To understand how the “Res-U-Net” works, we must understand first the residual blocks. The problem of vanishing gradients, especially at the first few layers after input of the network, causes the weights belonging to these layers not to be updated correctly during the backpropagation. However, more layers in the network means performance saturation and then a rapid decrease. Res-Net has proposed the identity matrix and the skip connection between layers. As a result of this identity matrix, the error signal can be backpropagated through these connections among the network, and the gradient will be multiplied by 1, which helps in preserving the input and reducing information loss.

**Figure 3:**
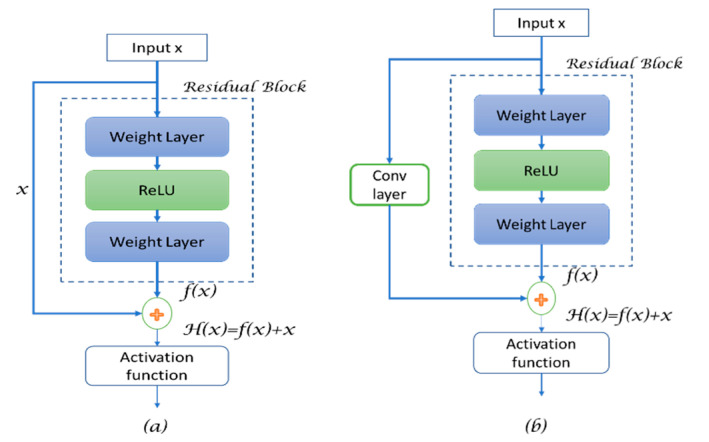
Residual block structure, (**a**) when the input is simple and it matches the output shape, (**b**) when the *X* shape does not match with the *f*(*x*) shape, hence a convolutional layer is added.

To understand how residual blocks work, let the input of the network be *x*. We assume that the final desired underlying mapping output is *f*(*x*), which further is fed into the activation function. The residual blocks take their name from the residual mapping between the output and input, which is *R*(*x*) = *H*(*x*) − *x*. Meanwhile, in the traditional neural network, the true output is *f(x*). However, after the residual mapping is learned, the addition between input (*x*) and the residual mapping (*R*(*x*)) is performed to present the final output *f*(*x*). It is worth noticing that adding the skip connection can take the compatibility between the added inputs into account, where the CNN output reduces the input size (dimensionally), thus, adding the input (*x*) is a problem. Therefore, it is important to add an operation or a function (convolutional function) to the skip connection to process the input so it can match the shape of *f*(*x*), as shown in Figure 3.

Since the weight layer *f*(*x*) tends to be a zero function, then *H*(*x*) tends to be the identity function, though the default function for such a network is the identity function.

Therefore, the definition of residual block can be updated to:

(4) $$Y=H(x)=F\left(x,\left\{w_{i}\right\}\right)+x$$

(5) $$Y=H\left(x\right)=F\left(x,\left\{w_{i}\right\}\right)+w_{s}x$$

where *X* and *Y* are the input and the output vectors of the layer considered, respectively, *w_i_* are the parameters within the CNN layer inside the dotted box, and *w_s_* are the configurations (e.g., convolution operation) to change the input shape to be identical to the output shape for the addition purpose. The dotted box refers to the residual block, while the solid line, which carries the input (*x*) to the addition operator, is called the residual connection.

In a normal deep network, each layer is fed into the next layer, while in Res-Net, each layer is fed into the next layer and is fed also to a further layer after some hops away, as the skip connections combine both the input and the output using an identity mapping operation where no additional parameters are needed.

**Figure 4:**
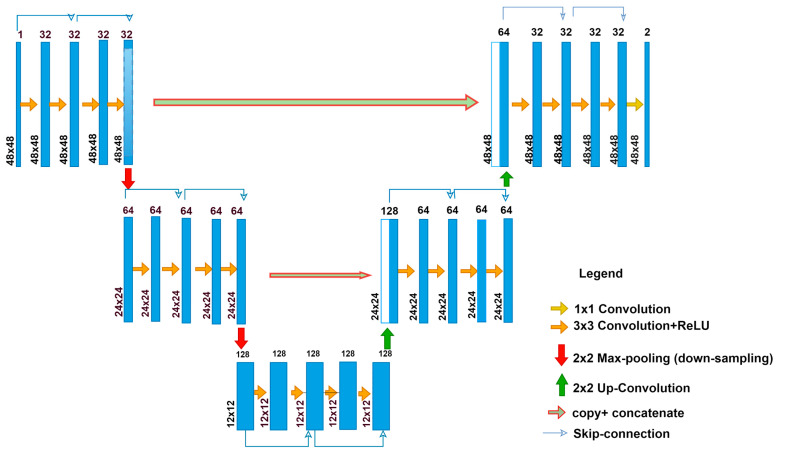
Residual U-Net architecture.

In summary, the Res-Net skip connection allows adding feature maps between a layer to other deeper layers of the network, which gives the network the ability to maintain feature maps in deeper networks to improve the performance for deeper networks. Residual U-Net is pictured in Figure 4. This addition of residual connections helped the basic U-Net to tackle the problem of vanishing gradients, and it gives the ability to use a deeper U-Net with more layers. From Figure 3b, we can denote the residual blocks as:

(6) $$Y^{\ell }=H\left(x\right)=h\left(x^{\ell }\right)+F\left(x,\left\{w_{i}\right\}\right)$$

(7) $$x^{\ell +1}=f(Y^{\ell })$$

where *Y^ℓ^* is the output of the layer after the residual block (the added output),

*R*(*X*) refers to the residual mapping,
*h*(*x*^ℓ^) is referred to as the identity map function after applying the convolution operation,
*x^ℓ^*^+1^ is the input for the next layer, and
*f*(.) is the activation function.

Much research in the medical image field has adopted the Residual U-Net for segmentation of breast cancer [38], brain structure mapping [39], and brain segmentation. In particular, this architecture was applied mostly for brain tumors and stroke analysis and segmentation, Zhang J et al. [40] have proposed Separable and Dilated Residual U-Net (SDResU-Net) for segmenting brain tumors from MR images. Their proposed method has captured more pixel-level details. Saeed Mu et al. [41] have used a hybrid DL model, which is Residual Mobile U-Net (RMU-Net), by modifying the MobileNetV2 model by adding residual blocks; this is further used as the encoder part in the U-Net model, while the decoder remains as the regular U-Net decoder. Authors have achieved good results on the BraTS (2018–2020) datasets for brain tumors. Other research that used Residual U-Net for brain tumors are found in [42,43].

### 2.4. Attention U-Net

After the new trait from image processing, which is the attention mechanism that focuses on a particular region within the image, which is the ROI, and ignores other areas of the image, this mechanism was implemented in many DL networks. Introducing this mechanism to the base U-Net architectures has produced the common new aspect, which is Attention U-Net [44]. To understand the Attention U-Net structure, we will go through the structure of the attention gate. An attention gate, in the context of segmenting images, is a method to focus only on the relevant activation during the training phase. Although, the major advantage is reducing the computational power consumption because it eliminates the irrelevant activations, which helps the network achieve a better generalization too.

**Figure 5:**
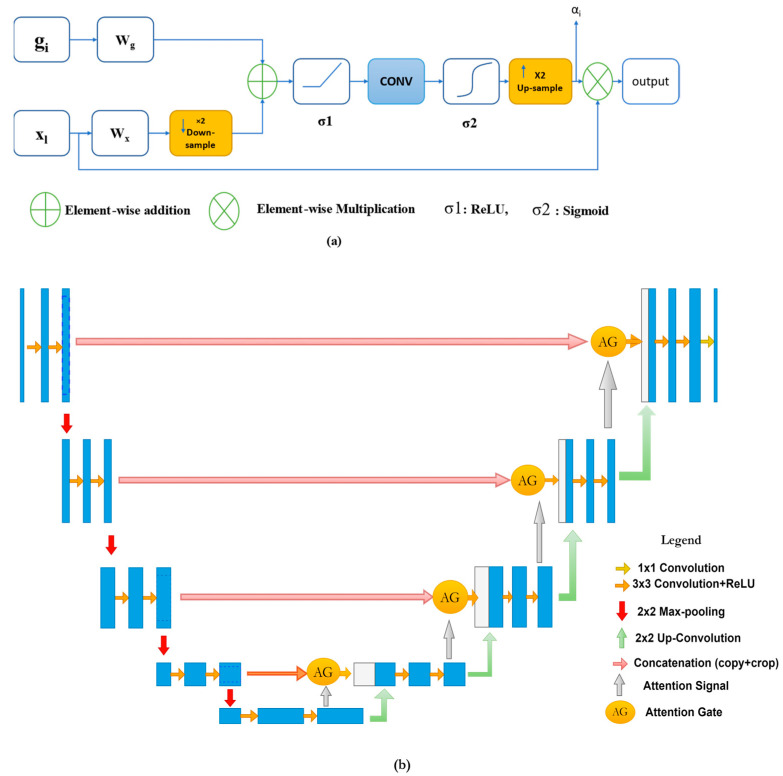
(**a**) Attention mechanism. (**b**) Attention U-Net architecture.

The typical structure of the attention gate is pictured in Figure 5a. Attention has two types, hard attention and soft attention. Whereas hard attention focuses only on one region at a time and is non-differentiable, the soft attention is differentiable and easier to train with backpropagation, moreover, it weights different parts of the image.

From Figure 5a, let *x_l_* be the feature map of the layer (*l*), gi is the gating signal from each pixel (*i*) to choose the region of interest, and *α_i_* is the attention coefficient (0< *α_i_* < 1), which is used to neglect the irrelevant regions and features while exporting the features that are relative to the targeted area. The final output (*x_out_*) is the element-wise multiplication between the input and the attention coefficients, defined by:

(8) $$x_{out}=x_{l}\cdot \alpha_{i}$$

Here, α*_i_* are the multi-dimensional coefficients used to only focus on a subset of the target image (cropped region), and it is given by:

(9) $$\alpha_{i}=\sigma_{2}(\psi^{T}\left(\sigma_{1}\left(W_{x}^{T}x_{l}+W_{g}^{T}g_{i}+b_{g}\right)\right)+b_{\psi })$$

where *σ*1 is the activation function (commonly ReLU), *σ*2 is the second activation function (commonly sigmoid activation function), *Wx*, *Wg*, and *ψ* are linear transformations, basically 1 × 1 channel-wise convolutional operations, and *b_g_* and *b_ψ_* are the biases terms for both the gating signal and the input *x*.

Oktay et al. [44] have also introduced a grid-based attention mechanism. In this type of attention mechanism, coefficients are more specific to a local region. In this type of gating, the signal is not a global vector for all the image pixels, but it is a grid signal dependent on the image spatial information. Moreover, the gating signal can aggregate features from multiple scales. The attention gate module parameters can be trained using regular backpropagation without needing the sampling approaches used in hard attention.

The attention gate has been used frequently in encoder–decoder deep networks. Especially in U-Net models, attention gates have been incorporated into U-Net to provide localized classification information as well as to improve the sensitivity and leverage the overall performance without performing significant computation, due to suppressing the irrelevant background regions. These modules have been implemented before the concatenation operation along with the skip connections between the compression path and the expansive one, although merging was only performed for relevant activation before up-sampling at the expansive path. This integration of these modules helps to down-weight the gradients from the background regions through the backpropagation update, therefore, the prior layers’ parameters are updated based on the spatial regions that are related to the given task (e.g., brain tumor segmentation). Vaswani A et al. [45] have illustrated that an attention gate uses a function by which it weights features’ maps corresponding to each class, which leads to focus on specific objects within an image. The most common attention type is the additive module, which provides more promising results in segmentation. The Attention U-Net basic structure is pictured in Figure 5b.

In biomedical image segmentation, Attention U-Net has been used for segmenting different body organs and diseases such as abdominal structure [46] and brain tissues segmentation [47].

### 2.5. Dense U-Net

As other architectures discussed in this paper, Dense U-Net has evolved by merging the successful networks together: Dense-Net [48] and the basic U-Net. The new modification that has been added to the base U-Net is using dense blocks instead of the convolutions at a regular layer. Dense-Net can reuse feature maps for improving the feature extraction performance. In addition, using dense blocks improves the accuracy of feature extraction and avoids re-using redundant features by comprising dense layers, residual layers, and transition layers too. Since Dense-Net has been built upon the Res-Net, but with some changes such that each layer receives the identity map from all the previous layers, where all identity maps (skip connections) are aggregated into tensors through channel-wise concatenation, here, Res-Net uses element-wise concatenation. This method promotes efficient gradient propagation. In medical images, to improve the segmentation performance, exploiting the features from different scales is required, for example, the low-level features extracted from the first few layers contain good spatial information, but they contain more noise and less semantic features. On the other hand, the high-level features have stronger semantic features with lower perception details and poor spatial information. Therefore, fusing dense blocks with different levels by using MFF block was proposed [49]. Dense-Net uses the same concept of the identity connections as Res-Net, but with the difference that each layer receives the feature maps from all the preceding layers. Equations below explain the concept of dense blocks. The regular CNN output of the lth layer is given by:

(10) $$x^{l}=H^{l}(x^{l-1})$$

where *x^l^* is the output of the lth layer, *x^l^*^−1^ is the output of the previous layer, and *H(x^l^*^−1^) is a convolution followed by non-linear activation function (e.g., ReLU) for the lth layer.

In Res-Net, the input is added to the output through an identity matrix (skip connection), so the equation becomes:

(11) $$x^{l}=H^{l}\left(x^{l-1}\right)+x^{l-1}$$

However, Dense-Net uses dense blocks, which exploit the skip connection concept as discussed above, where it uses all the preceding features maps in a feed forward scheme, and the equation becomes:

(12) $$x^{l}=H^{l}([x_{0},x_{1},\ldots ,x^{l-1}])$$

**Figure 6:**
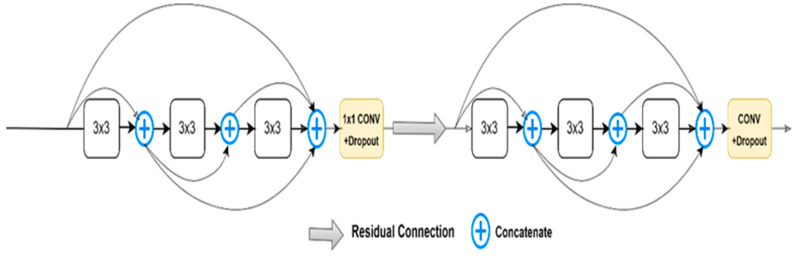
Dense connection blocks, 3 × 3 means convolution, in normal CNNs there are no skip connections between convolutional blocks.

Here, *H*(.) is defined as the composite function which has commonly sequential operations such as, batch normalization (BN), non-linear function (ReLU), and convolutional layer. The concatenation in dense blocks is channel-wise concatenation, as shown in Figure 6.

At any aggregation point, it will aggregate (k) feature maps by using a transition function for each layer. k is also referred to as the growth rate of the network, and it is responsible for the controlling of the contribution of information corresponding to each layer to the whole network’s feature maps.

**Figure 7:**
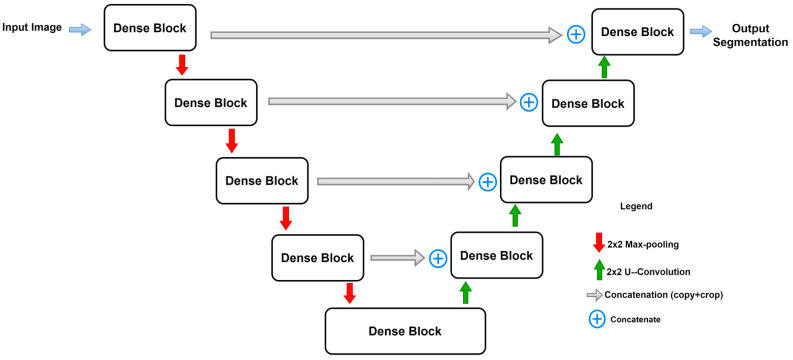
Dense U-Net structure, dense blocks are implemented instead of U-Net normal convolutions.

Transition functions are used between the dense blocks within a layer called the transition layer, and this layer is responsible for the concatenating of feature maps. There are two types of transitions (transition down, and transition up). Transition down contains consecutive operations such as BN, ReLU, (1 × 1) convolution, and average pooling layer, while the transition up contains 2 × 2 up-sampling. Dense U-Net is shown in Figure 7.

Kolarik M et al. [30] have used 3D Dense U-Net for brain MRI super-resolution. An attention gate was also introduced for Dense U-Net for breast mass segmentation in digital mammograms [50] and for brain lesion segmentation [51]. 3D Dense U-Net was also proposed for segmenting brain tissues [52] and lesion and spine segmentation [53].

### 2.6. U-Net++

**Figure 8:**
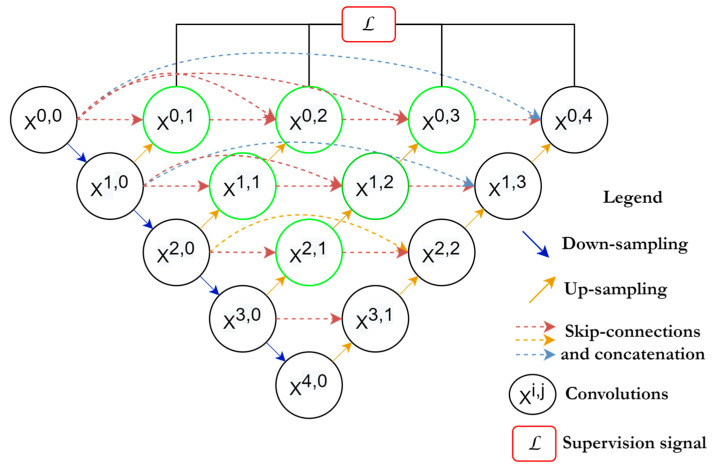
Basic U-Net++ architecture.

U-Net++ [54] is inspired by Dense-Net. The outfit scheme of this network involves an intermediary grid block between the contracting and the expanding path and using dense blocks and connections in between. These intermediary blocks help the network to transfer more semantic segmentation between the regular paths as well as increase the accuracy of segmentation. As is shown in Figure 8, every unit receives the feature maps from the units at the same level, plus the up-sampled feature maps from the exact lower layer units. Hence, units at the same level are all densely connected, furthermore, units at the lower level are connected through skip connections towards the upper layer’s units. The idea behind using the in-between densely connected convolutional blocks is that these blocks ensure the semantic feature maps from the encoder are more similar to those at the decoder, which will help the network optimizer to optimize the network more efficiently when these feature maps are more similar semantically between the encoder and the decoder. According to the original paper [54], the pathway of the skip connections among both paths are arranged considering that x^i,j^ is the output of the node, X^i,j^ and i,j are the indices of the down-sampling layers at the encoder and the indices of the convolutional layer of the dense block at the same level. The operation of aggregating the feature maps received at each unit is given by:

(13) $$x^{i,j}=\left\{\begin{array}{c} H\left(x^{i-1,j}\right),j=0\\ H\left(\left[{\left[x^{i,k}\right]}_{k=0}^{j-1},U\left(x^{i+1,j-1}\right)\right]\right),j>0 \end{array}\right.$$

Here, *H*(.) is referred to as the convolutional operation followed by the ReLU activation function, *U*(.) is the up-sampling operation, which contains the up-convolution operations, and [.] is the concatenation process. The first row of U-Net++ units (*j* = 0) receive their dense inputs only from the preceding layer belonging to the encoder at the same level (*j* = 0).

Meanwhile, the rest of the rows (e.g., *j* = 1) receive two inputs, first from the preceding layers at the same level and second from the lower layer (*j* = 2) where this input is an up-sampled output of the lower skip pathway.

U-Net++ is also mainly used for medical image segmentation for different organs in the body.

A. Hou et al. [55] have used it for brain tumor segmentation, and Micallef. N et al. [56,57] have used this architecture for brain tumors too, and other applications such as liver cancer [54,58].

### 2.7. U-Net 3+

**Figure 9:**
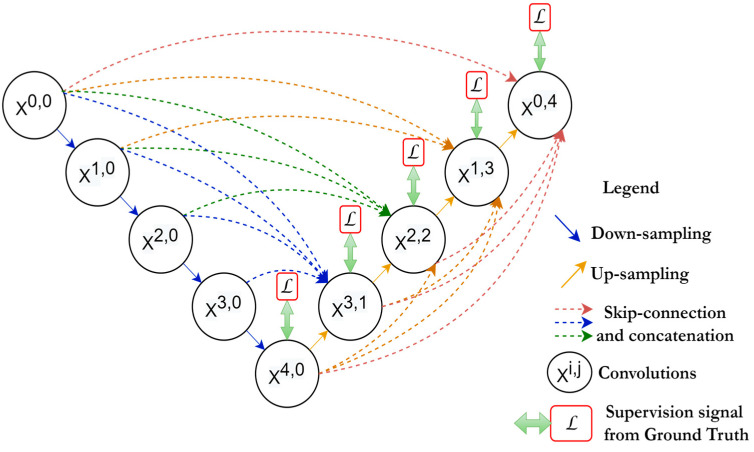
U-Net 3+ architecture.

U-Net 3+ is another variant of U-Net and is more similar to the U-Net++ architecture with some minor changes in architecture structure [59] is shown in Figure 9. Dense skip connections connect the contracting and expansive paths. U-Net 3+ benefits from full-scale connections and deep supervision, with each decoder layer receiving signals from the encoder and decoder. Deep supervision learns hierarchical representations from feature maps, and a classification-guided module is added to address noisy background information. Comparing to the U-Net++ model, U-Net 3+ reduces the parameters for efficient computation. In addition, the authors of [59] have compared this network to U-Net and U-Net++ on two datasets, the first one is for liver segmentation (ISBI LiTs 2017) and another is for spleen segmentation (locally collected dataset). Their network outperformed both other networks.

### 2.8. Adversarial U-Net

Since Generative Adversarial Networks (GANs) have been introduced by Ian Goodfellow in 2014 [60], they have received big attention in later research. They were first used to generate new data, by which two CNN networks are competing against each other so both can learn and improve. The two major networks are called the generator (G) and the discriminator (D), where (D) receives two inputs and it must classify whether this input is real or fake (received from the generator) and the generator produces images from noise input, which produce variations of images. The discriminator network is a standard supervised learning type CNN, it produces the probability of an image being generated by (G), and it tries to minimize the error when classifying fake images as real dataset images, and this is where the generator outperforms the discriminator. To train the generator for producing closer images to the real ones, we make the generator gradient function as a function of the discriminator’s gradient function. In this way, the generator learns to adjust its weights according to the discriminator output. The adversarial concept came from the fact that the generator is trying to deceive the discriminator and increase its error rate.

The generator learns the mapping from the random noise vector (*z*) and finally produce the image (*x_g_*)

$$x_{g},\;\;G:z\to x_{g}$$

where *G* is the generator and *D* is the discriminator.

The relationship between the generator and discriminator is given by:

(14) $$minmaxV\left(D,G\right)=E_{{x\sim }_{Pdata}\left(x\right)}\log D\left(x\right)+E_{{z\sim }_{Pz}\left(z\right)}\log\left(1-D\left(G\left(z\right)\right)\right)$$

At the last phase of the network training, the discriminator will not differentiate the real images from the fake ones (synthetic) generated by the generator. The new generated images will be considered as artificial images, and they can be used for creating a new dataset for a specific subject.

Since the images generated by GANs are randomized and difficult to assign labels, conditional GANs [61] are introduced to tackle this problem. Conditional GANs take the random noise vector (*z*) and observed images *x_i_* for a specific class c_t_ to the generated images *x_g_*, *G_c_*: (*z*, *x_i_*) →*x_g_.* GANs are designed upon the discrepancy measurement between the generated data and the real data. The objective function or the minmax relationship among the generator and the discriminator is given by:

(15) $${ℒ}_{c}\left(G_{c},D\right)=E_{x_{i},x_{g}}\left[\log D(x_{i},x_{g}\right)+E_{x_{i},z}\left[\log(1-D(x_{i},G_{c}(x_{i},z\right)))]$$

where *G_c_* tries to minimize the objective function while the discriminator *D* tries to maximize it (that is why it is called the minmax relationship). It is shortly denoted as:

(16) $$G=\arg\underset{G_{c}}{\min}\underset{D}{\max}{ℒ}_{c}\left(G_{c},D\right)$$

**Figure 10:**
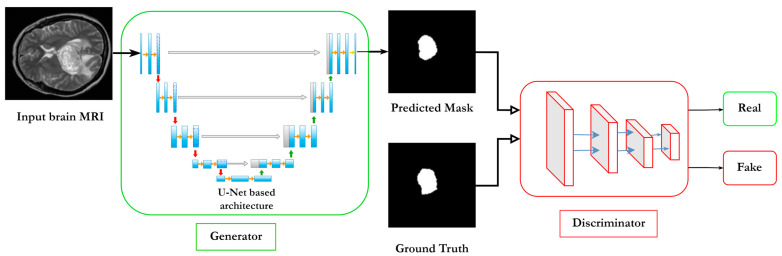
Simplified schematic of the Adversarial U-Net structure. The generator network is represented by basic U-Net architecture.

Adversarial U-Net has both the basic architectures of a conditional GAN and the basic U-Net, where the U-Net architecture is implemented in the generator, while the discriminator remains the same network. The key term of using U-Net architecture in the generator is to generate transformed images, where the generator input is no longer a noise, but an image waiting to be transformed. The discriminator is trained manually on transformed images, and it is responsible for evaluating the generator output. In summary, the generator is trained to produce transformed images, in other words, it learns the transformation required function to produce transformed images the same as the manual human transformation process. Then, the transformation process is automated, and after the training is done, the generator is used to do the work, in this way, faster transformation is being done in a faster fashion than a physician manually converting the images. An illustration of Adversarial U-Net where U-Net structure is used at the generator is pictured in Figure 10.

Chen X et al. [62] have used Adversarial U-Nets for domain-free medical image augmentation. U-Net also was used for both the generator and discriminator here.

Adversarial U-Net has been applied for various tasks regarding medical images, such as image registration of brain structure [63], brain tumor detection [64], brain quantitative susceptibility [65], and brain tumor segmentation [66].

### 2.9. Other Well-Known Architectures Based on U-Net

In the preceding sections, we have discussed the most used variants of U-Net since its invention, but there are many more architectures based on it. We will only mention some other popular optimizations, which have been inspired by U-Net, used for medical images segmentation, and many of these architectures were built upon each other or merged to obtain the advantages from each other. Here are some of these architectures, Trans U-Net [67], V-Net [68], Recurrent U-Net [69], Ensemble U-Net [70,71], Kiu-U-Net [72], Attention Residual U-Net [73].

## 3. Materials and Methods

### 3.1. Loss Functions

Optimization methods do not involve architecture modeling only, but they also include loss functions and activation functions too. Loss functions are categorized into different categories (e.g., distribution-based loss, region-based loss, boundary-based loss, and compound loss).

#### 3.1.1. Cross-Entropy Loss

Here, we are briefly demonstrating some commonly used loss functions used for medical image segmentation. The most used loss function is cross-entropy loss [74], and it is derived from Kullback–Leibler (KL) divergence to evaluate the variation (dissimilarity) between two distributions. It is given by:

(17) $$L_{ce}=-\sum_{i-1}^{N}\left[g_{i}\log\left(p_{i}\right)+\left(1-g_{i}\right)\log\left(1-p_{i}\right)\right]$$

where *p_i_* refers to the training result, *g_i_* refers to the ground truth, and *N* is the number of pixels. Cross-entropy loss converges quickly because the gradient of the last layer is not relevant to the activation function, where the difference is only related to the result and the ground truth. Many researchers use cross-entropy loss, but using this loss is preferable when the segmented target is not extremely different from the background. However, region-based loss is more likely to be used when this type of loss aims to minimize the mismatch or maximize the overlapping between the segmentation results and the ground truth.

#### 3.1.2. Dice Loss Function

Another widely used loss function is the Dice loss function, used for medical image segmentation. It is extracted from the Sorensen–Dice coefficient [75], and it directly optimizes the mostly used metric for segmentation, which is the Dice coefficient. The Dice loss is given by:

(18) $$D=\frac{2\sum_{i}^{N}p_{i}g_{i}}{\sum_{i}^{N}p_{i}^{2}+\sum_{i}^{N}g_{i}^{2}},\;\in [0,1]$$

Here, *g_i_* is the ground truth pixels (voxels if 3D segmentation task) and *N* is the number of pixels. Since, in image segmentation networks, the last layer, which is mostly a SoftMax layer, the output is a probability of each pixel belonging to foreground or background. In addition, the Dice loss can be differentiated to produce the gradient:

(19) $$\frac{\partial D}{\partial p_{j}}=\frac{2\left|GT\cap Pr\right|}{\left|GT\right|+\left|Pr\right|}=2\left[\frac{g_{i}(\sum_{i}^{N}p_{i}^{2}+\sum_{i}^{N}g_{i}^{2})-2p_{i}(\sum_{i}^{N}p_{i}g_{i})}{{\left(\sum_{i}^{N}p_{i}^{2}+\sum_{i}^{N}g_{i}^{2}\right)}^{2}}\right]$$

The aim of Dice loss is to establish the right balance between the foreground (target) and the background by tuning the weight matrices.

Another extension of Dice loss is the generalized Wasserstein Dice loss [76] used for multi-class segmentation, which takes the advantages of the hierarchal structure of complicated tissues.

#### 3.1.3. IoU Loss

Intersection over union [77], or Jaccard loss, is identical to Dice loss and belongs to the same category of region-based loss. It is derived from the Jaccard index, and it simply measures the intersection between the segmentation results and the ground truth. It is given by:

(20) $$IoU=\frac{\left|GT\cap Pr\right|}{\left|GT\cup Pr\right|}=\frac{\left|GT\cap Pr\right|}{\left|GT\right|+\left|Pr\right|-\left|GT\cap Pr\right|}=\frac{\sum_{i}^{N}p_{i}g_{i}}{\sum_{i}^{N}p_{i}^{2}+\sum_{i}^{N}g_{i}^{2}},\in [0,1]$$

*GT* is the ground truth. *P_r_* is the output segmentation result.

#### 3.1.4. Tversky Loss

This loss is also a region-based loss and is a modified Dice loss. It sets different weights to the false negative (FN) and false positive (FP), whereas Dice loss uses the same weights for the preceding terms. This makes Tversky loss suitable for the unbalanced datasets. The Tversky loss formula is given by:

(21) $$T_{l}=\frac{\left|GT\cap Pr\right|}{\left|GT\cap Pr\right|+\alpha \left|Pr\backslash GT\right|+\beta \left|GT\backslash Pr\right|}$$

It is also formulated as:

(22) $$T\left(\alpha ,\beta \right)=\frac{\sum_{i=1}^{N}p_{ic}g_{ic}}{\sum_{i=1}^{N}p_{ic}g_{ic}+\alpha \sum_{i=1}^{N}p_{i\overset{-}{c}}g_{ic}+\beta \sum_{i=1}^{N}p_{ic}g_{i\overset{-}{c}}}$$

where *p_ic_* is the probability that pixel *i* is from class *c*, $p_{i\overset{-}{c}}$ is the probability that pixel *c* is not from the class *c* (for example, class c means tumor tissue), and same terminology applies for *g_ic_* and $g_{i\overset{-}{c}}$ considering it is ground truth pixels. *α* and *β* are the hyperparameters, and tuning these two parameters can shift the emphasis to better the recall when having class imbalance [78].

#### 3.1.5. Hausdorff Distance Loss

Finally, the boundary losses category aims to minimize the distance between both the segmentation result and the ground truth [79]. It is used for extremely unbalanced data, and the most used boundary loss function is Hausdorff distance loss, which tries to estimate the Hausdorff distance from the network output probability and aims to reduce it. Hausdorff distance loss is given by:

(23) $$L_{HD}=\frac{1}{N}\sum_{i=1}^{N}\left[\left(p_{i}-g_{i}\right)\circ \left(d_{Gi}^{2}+d_{pi}^{2}\right)\right]$$

where *d_Gi_* and *d_pi_* are the distances of the ground truth and segmented result, respectively and o is the Hadamard Product (entry-wise).

Lastly, compound loss functions are used by summing over various types of loss functions to produce new mixed loss functions.

### 3.2. Evaluation Metrics

Choosing the right metric for evaluating any task in deep learning is vital because specific metrics are used to evaluate different tasks. In this section, we will briefly present the widely used metrics for medical image segmentation. Rather than elaborating about the evaluation metrics used for different tasks that use deep learning for medical image analysis, we will only focus on the metrics for segmentation.

#### 3.2.1. Dice Coefficient

The first and the most common metric for validating medical volume segmentation is called Dice-score coefficient (DSC) (or overlap index) [75]. This metric is defined by Equation (24):

(24) $$DICE=\frac{2\left|S_{g}\cap S_{p}\right|}{\left|S_{g}\right|+\left|S_{p}\right|}=\frac{2TP}{2TP+FP+FN},\in [0,1]$$

*S_g_* and *S_p_* are the segmented region of ground truth and the predicted segmentation result, respectively. The value of DICE is “0” when there is no overlapping between the resulting segmented area and the ground truth segmented area, and it is equal to “1” when they are 100% overlapped. Since the confusion matrix calculates all the distribution probabilities, many evaluation metrics are derived from the terminologies corresponding to the confusion matrix, such as true positive (TP), true negative (TN), false positive (FP), and false negative (FN).

#### 3.2.2. Jaccard Index/Intersection over Union (IoU)

IoU [77] calculates overlapping area between the ground truth and the segmentation result divided by their union. Therefore, it gives an idea about the similarity between both regions. It is given by the formula:

(25) $$JAC=IoU=\frac{\left|S_{g}\cap S_{p}\right|}{\left|S_{g}\right|+\left|S_{p}\right|}=\frac{TP}{TP+FP+FN},\;\in [0,1]$$

From the equation above, we note that the difference between DICE and IoU is that IoU is always greater than DICE, except at the peak {0,1} where they are equal. In addition, the relation between both metrics is given by:

(26) $$JAC=IoU=\frac{\left|S_{g}\cap S_{p}\right|}{\left|S_{g}\right|+\left|S_{p}\right|}=\frac{2\left|S_{g}\cap S_{p}\right|}{2(\left|S_{g}\right|+\left|S_{p}\right|-\left|S_{g}\cap S_{p}\right|}=\frac{DICE}{2-DICE}$$

Similarly

(27) $$DICE=\frac{2JAC}{1+JAC}$$

Which means that both metrics measure the same aspects and evaluate the system ranking, hence, selecting one metric to evaluate the results is enough.

#### 3.2.3. Hausdorff Distance (HD)

It is one of the recent rising used metrics for evaluation of a segmentation task, however, reducing the Hausdorff distance is the goal of segmentation because it is evidence of the segmentation error. For two-point sets, *X* and *Y*, the distance from *X* to *Y* is defined as:

(28) $$HD\left(X,Y\right)=\frac{1}{N}\sum_{x\in X}\underset{y\in Y}{\min}\left\|x-y\right\|$$

where *N* is the total number of observations (voxels or pixels).

Moreover, the average Hausdorff distance between *X* and *Y* is given by:

(29) $$d_{AHD}\left(X,Y\right)=\left(\frac{1}{X}\sum_{x\in X}\underset{y\in Y}{\min}d\left(x,y\right)+\frac{1}{Y}\sum_{y\in Y}\underset{x\in X}{\min}d(x,y)\right)/2$$

Therefore, the average Hausdorff distance can be calculated as the mean of the directed average from *X* to *Y* and from *Y* to *X*.

For the medical image segmentation, we assume that point set *X*, and point set *Y* are the ground truth voxels and the segmentation result voxels, respectively. Therefore, the HD can be calculated in millimeters or voxels, then Equation (29) can be written as:

(30) $${HD}_{avg}=(\frac{G\;to\;S}{G}+\frac{S\;to\;G}{s})/2$$

where *G* to *S* is the directed average *HD* from the ground truth to the segmentation result, and vice versa for the term *S* to *G*, where *G* and *S* are the voxels of the ground truth and the segmentation result, respectively. *HD* is sensitive to outliers.

#### 3.2.4. Sensitivity and Specificity

Also called true positive rate (TPR) or recall, this metric measures the positive pixels fraction in the ground truth, which also are predicted as positive in the segmented result. Similarly, true negative rate (TNR) or specificity gauges the negative pixels (background) that are identified as negative pixels from the ground truth and the segmentation result. These two metrics are both valuable because of their sensitivity to the segment sizes, which make them suitable for segmenting small size regions (e.g., retina vessels) because they penalize the small segments [80]. We demonstrate the formula of sensitivity, and specificity as:

(31) $$Recall=Sensitivity=TPR=\frac{TP}{TP+FN}$$

(32) $$Specificity=TNR=\frac{TN}{TN+FP}$$

The preceding metrics are the major metrics used for medical image segmentation, and there are other evaluation metrics, but they less common, which are highlighted in [81,82].

### 3.3. Comparison and Analysis

After reviewing the major deep learning architectures, we provide an analytical perspective of the performance of these DL models against brain tumor segmentation. Table 1 shows the performance of models based on U-Net against brain tumor segmentation. We have covered the discussed models above in this table that were applied for the BraTS-2020 [83] challenge to be more precise about the evaluation against a standard unified dataset such as BraTS. In general, it was found from the literature that evaluating deep learning models for medical image segmentation requires all the numerous configurations related to deep learning (e.g., optimizer, loss function, hyperparameters, etc.) to be fixed. Table 1 shows comparatively slight changes corresponding to the Dice score metric. [84] have demonstrated that a simple U-Net can outperform more complex model such as the adversarial-based U-Net architectures for segmentation if the model network is optimized and well-tuned. For instance, the same base Attention U-Net variant has shown a comparative difference in DSC, whereas [85], in their model, have shown low DSC values compared to [86], which have used the same model architecture with slight changes.

## 4. Experimental Results

We have conducted experimental work by using mainly four U-Net architectures. Our experimental work uses the MICCAI BraTS 2020 challenge dataset, which includes 369 examples for training, whereas the validation dataset contains 125 samples.

### Experimental Training Layout

The training dataset was split into 80% for training (295 MRIs) and 20% for validation (74 MRIs), where the 4 modalities were used to generate the 4-channel volume. The labels provided by the dataset (ET, NET-NCR, ED) were converted into 3-channel volume and labeled as enhanced tumor (ET), tumor core (TC), and whole tumor (WT). The generic flow of pre-processing was followed in our experiments using the Medical Open Network for Artificial Intelligence (MONAI) framework, where all MRIs are cropped to a smaller size to minimize the computation’s need, and the output volume has (128 × 128 × 128) dimensions.

It was found that after epoch 200, the Dice score did not improve, so we considered it as the main number of epochs.

Our results were evaluated on the challenge validation dataset using the submission portal (https://ipp.cbica.upenn.edu/) accessed on 15 December 2022. The experimental setup and configurations are as follows:

Ubuntu 20.04, NVIDIA RTX A6000 48GB memory and 197 GB of RAM, where the software used are python 3.9 and cuda 11.3.

We have used the same hyperparameters for all used models. The U-Net architectures used are:

- 3D U-Net: This architecture consists of four levels of convolutions in both the encoder and decoder. It was proposed in [96].
- Modified 3D U-Net: follows the same attributes as the previous model, but an extra level is added, so the encoder–decoder network uses five levels of convolutions.
- Attention U-Net: [44] similar to the 3D U-Net model as the main architecture, but attention gates are used as shown in Figure 6 at the decoder side.
- R2 Attention U-Net: Recurrent Residual Attention U-Net was proposed in [97], which adds the recurrent and residual blocks to the first 3D model.

The main hyperparameters and attributes used are included in Table 2.

The segmentation results achieved for the 4 models are shown in Table 3, where the time needed for training on the 80% of training dataset (295) sample is determined for 200 epochs, and the time needed for 1 sample is listed too.

**Figure 11:**
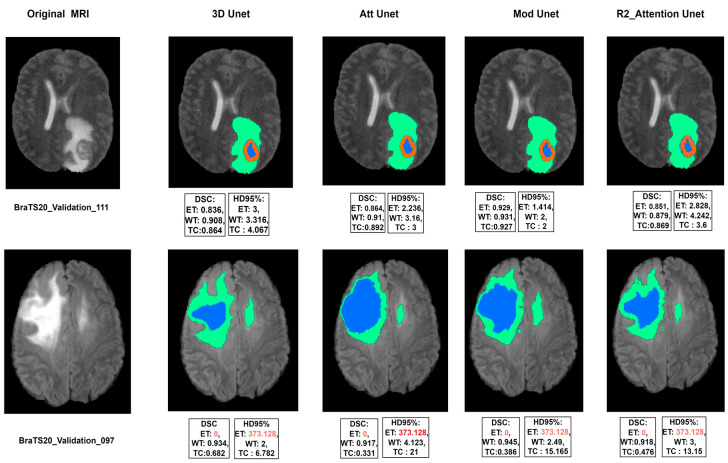
Good (**top**) and bad (**bottom**) segmentation results achieved by the four models, where the green, red, and blue colors represent the brain tumor subregions of whole tumor (WT), enhanced tumor (ET), and tumor necrotic core (TC) respectively.

Our experimental work showed slight changes in Dice score and Hausdorff distance, however, time needed for training and the number of parameters used for these models are different. A demonstration of the visual results of the validation dataset achieved by the four experimented models is pictured in Figure 11, where the numerical results included have been evaluated through the challenge portal. It was found that a bad segmentation performance is correlated to the absence of one or two labels in the validation dataset.

## 5. Discussion

The original approach to medical image segmentation, in general, and brain tumor segmentation, in particular, is heading towards optimization in terms of different aspects of deep learning, where model architecture is one of these aspects. More complex models were found to be not efficient in general [84], for instance, the adversarial segmentation approach requires more computational power because instead of one network, two networks are used for training, although the performance is still within the same range as simpler models. Eventually, it becomes clear from Table 1 and Table 3 that U-Net-based models provide state-of-the-art results with slight differences, therefore, other optimization approaches are used as extensions for such models, and such optimizations are represented by using different optimizers and loss functions.

The optimization methods that were added after U-Net have exploited the high-level design and produced even more accurate results and maintained better performance. The interesting thing about U-Net is that it can be used for a wide spectrum of applications because of the ability for tuning and adjusting according to different applications. Moreover, the modular nature of this architecture allows it to be able to improve, and this is what we have seen from incorporating different architectures with it and novel optimization methods, which increased its robustness. We have mainly focused on brain MRI segmentation. Recently, U-Net and its robust models have become available and are easy to implement through different programing frameworks as packages, for example, keras-unet-collection, which contain the base U-Net architecture and a few other architectures (ResU-Net, Attention U-Net, U-Net 3+, U-Net++, 3D U-Net, etc.).

Fine tuning the network architecture along with other parameters (loss functions, optimizers, normalization layers, and other blocks) aims to optimize network performance. For instance, DeepLab is a segmentation model that involves atrous spatial pyramid pooling (ASPP) [98] to allow the network to extract contextual features at different scales without increasing the number of parameters, which minimizes the computations. Moreover, ensemble models are widely used to combine the benefits and improve the overall performance. 

### 5.1. Limitations of this Research

U-Net-based models have a complex architecture and require a large number of computational resources, which can make them difficult to implement using normal machines. In addition, training such networks, especially the generative-based models, is a time-consuming task [99].

Due to the high-dimensional nature of medical images, U-Net-based models may be prone to overfitting, particularly when training on small datasets. This can lead to poor generalization performance on new unseen data.

In summary, U-Net-based models for brain tumor segmentation are affected by limitations in data availability, class imbalance, and generalization. However, these limitations can be addressed by using advanced techniques such as data augmentation [100], regularization [101], and ensemble methods and using more sophisticated architectures [96].

### 5.2. Challenges

The existence of small labeled medical datasets for training is one of the common important challenges for most deep learning models, but some optimized tools were used, such as exploiting the 3D volumetric data, since they have redundant information. Another solution was proposed in the U-Net-based paper [25] by applying random deformation to generate new samples.

Another way to increase the dataset size is by using generative learning methods such as GANs for synthesizing new annotated medical data [102].

Ultimately, one of the most vital challenges is the curious behavior of deep learning models, because the internal structure of deep networks is complicated and still usually empirically adjusted, such as tuning the hyperparameters and selecting suitable activation functions, loss functions, and number of hidden layers. In addition, due to these challenges deep learning is still facing, less dependency and accountability can be applied for large-scale real-world medical field applications since these applications are critical and not amenable for errors [103]. To leverage the benefits of deep learning in medical image segmentation, new methodology consists of combining the advantages of model-driven techniques, architectures, and categories of learning (supervised and un-supervised) to produce hybrid and optimized methods. Despite all the challenges and limitations, deep learning is still developing and being optimized in the medical field and is expected to be irreplaceable in the future.

## 6. Conclusions

In this paper, we have provided a close-up overview of the extraordinary deep learning architecture “U-Net” and its top variants used for brain tumor segmentation. The significance of having an optimal deep learning model lies in the need for an accurate method of segmentation for medical data. Most recent research is based on U-Net models rather than other deep learning models. Therefore, we have provided a quantitative comparison between multiple U-Net models found in the literature and another experimental comparison to understand which models perform better than others. We discussed limitations and challenges associated with using U-Net-based models for brain MRI segmentation for future scope research. To overcome these challenges, future research should focus on developing advanced techniques such as data augmentation, regularization, ensemble methods, and more sophisticated architectures and interpretable models. Eventually, deep learning will not replace radiologists, instead, it will aid them in diagnostics, and a combination of radiologists and deep learning models will improve the performance and accuracy in medical field applications.

## Figures and Tables

**Table 1:** A comparative analysis of main variants of U-Net architecture for brain tumor segmentation.

Ref.	Model	DSC		
		ET	WT	TC
[87]	Modified U-Net	0.7412	0.8988	0.8086
[88]	HI-Net	0.741	0.906	0.842
[89]	Vox-to-vox	0.75	0.892	0.791
[41]	Residual Mobile U-Net	0.832	0.913	0.881
[84]	nnU-Net architecture with augmentation and modification	0.82	0.889	0.85
[90]	Dense U-Net	0.791	0.891	0.847
[91]	Attention 3D U-Net	0.78	0.92	0.87
[92]	Residual U-Net	0.82	0.86	0.84
[93]	Inception Residual Dense Nested U-Net	0.819	0.88	0.876
[94]	Cascaded 3D Dense U-Net	0.78	0.901	0.83
[95]	Trans U-Net (TransBTS)	0.787	0.909	0.817
[68]	Deep V-Net	0.689	0.861	0.779

**Table 2:** Hyperparameters and configurations used for experimented models.

Activation Function	Leaky-ReLU
Epochs	200
Loss function	Dice loss
Optimizer	Adam

**Table 3:** Segmentation results on the BraTS 2020 validation dataset for the four experimented models.

Model	DSC			HD95%			Parameters	Time
	ET	WT	TC	ET	WT	TC		
3D U-Net [96]	0.779	0.881	0.827	27.23	7.788	8.278	23 M	6 h (1.2 s/sample)
Modified U-Net	0.781	0.905	0.807	26.607	5.785	18.545	26 M	10 h (3.8 s/sample)
Attention U-Net [44]	0.778	0.878	0.827	26.662	7.794	8.305	23.2 M	6.2 h (1.7 s/sample)
R2 Attention U-Net [97]	0.7426	0.8784	0.7993	36.653	9.228	9.95	22 M	5.8 h (0.8 s/sample)

## Data Availability

The dataset used in this work for the experimental results and to support the findings is publicly available from the MICCAI BraTS 2020 challenge, which can be acquired from https://ipp.cbica.upenn.edu/. Access date on 15 December 2022.

## References

[1] Munsif M., Ullah M., Ahmad B., Sajjad M., Cheikh F.A. (2022). Monitoring Neurological Disorder Patients via Deep Learning Based Facial Expressions Analysis. Artificial Intelligence Applications and Innovations. AIAI 2022 IFIP WG 12.5 International Workshops.

[2] Hussain A., Khan A., Yar H. Efficient Deep learning Approach for Classification of Pneumonia using Resources Constraint Devices in Healthcare. Proceedings of the 5th International Conference on Next Generation Computing.

[3] Li J.P., Khan S., Alshara M.A., Alotaibi R.M., Mawuli C. (2022). DACBT: Deep learning approach for classification of brain tumors using MRI data in IoT healthcare environment. Sci. Rep..

[4] Chopra P., Junath N., Singh S.K., Khan S., Sugumar R., Bhowmick M. (2022). Cyclic GAN Model to Classify Breast Cancer Data for Pathological Healthcare Task. Biomed Res. Int..

[5] Haq A.U., Li J.P., Khan I., Agbley B.L.Y., Ahmad S., Uddin M.I., Zhou W., Khan S., Alam I. (2022). DEBCM: Deep Learning-Based Enhanced Breast Invasive Ductal Carcinoma Classification Model in IoMT Healthcare Systems. IEEE J. Biomed. Health Inform..

[6] Agbley B.L.Y., Li J.P., Haq A.U., Bankas E.K., Mawuli C.B., Ahmad S., Khan S., Khan A.R. (2023). Federated Fusion of Magnified Histopathological Images for Breast Tumor Classification in the Internet of Medical Things. IEEE J. Biomed. Health Inform..

[7] Haq A.U., Li J.P., Ahmad S., Khan S., Alshara M.A., Alotaibi R.M. (2021). Diagnostic approach for accurate diagnosis of COVID-19 employing deep learning and transfer learning techniques through chest X-ray images clinical data in E-healthcare. Sensors.

[8] Lu S.-Y., Zhang Z., Zhang Y.-D., Wang S.-H. (2022). CGENet: A Deep Graph Model for COVID-19 Detection Based on Chest CT. Biology.

[9] Khan J., Khan G.A., Li J.P., AlAjmi M.F., Haq A.U., Khan S., Ahmad N., Parveen S., Shahid M., Ahmad S. (2022). Secure smart healthcare monitoring in industrial internet of things (iiot) ecosystem with cosine function hybrid chaotic map encryption. Sci. Program.

[10] Fazil M., Khan S., Albahlal B.M., Alotaibi R.M., Siddiqui T., Shah M.A. (2023). Attentional Multi-Channel Convolution With Bidirectional LSTM Cell Toward Hate Speech Prediction. IEEE Access.

[11] Khan S., Fazil M., Sejwal V.K., Alshara M.A., Alotaibi R.M., Kamal A., Baig A.R. (2022). BiCHAT: BiLSTM with deep CNN and hierarchical attention for hate speech detection. J. King Saud Univ. Inf. Sci..

[12] Khan S., Kamal A., Fazil M., Alshara M.A., Sejwal V.K., Alotaibi R.M., Baig A.R., Alqahtani S. (2022). HCovBi-Caps: Hate Speech Detection Using Convolutional and Bi-Directional Gated Recurrent Unit With Capsule Network. IEEE Access.

[13] Morrow M., Waters J., Morris E. (2011). MRI for breast cancer screening, diagnosis, and treatment. Lancet.

[14] Zhao M., Cao X., Zhou M., Feng J., Xia L., Pogue B.W., Paulsen K.D., Jiang S. (2022). MRI-Guided Near-Infrared Spectroscopic Tomography (MRg-NIRST): System Development for Wearable, Simultaneous NIRS and MRI Imaging. Multimodal Biomedical Imaging XVII.

[15] Kirkham A.P.S., Emberton M., Allen C. (2006). How Good is MRI at Detecting and Characterising Cancer within the Prostate?. Eur. Urol..

[16] Kasivisvanathan V., Rannikko A.S., Borghi M., Panebianco V., Mynderse L.A., Vaarala M.H., Briganti A., Budäus L., Hellawell G., Hindley R.G. (2018). MRI-Targeted or Standard Biopsy for Prostate-Cancer Diagnosis. N. Engl. J. Med..

[17] Singh A.K., Khan I.R., Khan S., Pant K., Debnath S., Miah S. (2022). Multichannel CNN model for biomedical entity reorganization. BioMed Res. Int..

[18] Prasoon A., Petersen K., Igel C., Lauze F., Dam E., Nielsen M. (2013). Deep Feature Learning for Knee Cartilage Segmentation Using a Triplanar Convolutional Neural Network. Medical Image Computing and Computer-Assisted Intervention—MICCAI 2013.

[19] Siddique N., Paheding S., Elkin C.P., Devabhaktuni V. (2021). U-Net and Its Variants for Medical Image Segmentation: A Review of Theory and Applications. IEEE Access.

[20] Lu S., Wang S.-H., Zhang Y.-D. (2021). Detection of abnormal brain in MRI via improved AlexNet and ELM optimized by chaotic bat algorithm. Neural Comput. Appl..

[21] Gordillo N., Montseny E., Sobrevilla P. (2013). State of the art survey on MRI brain tumor segmentation. Magn. Reson. Imaging.

[22] Recht M.P., Dewey M., Dreyer K., Langlotz C., Niessen W., Prainsack B., Smith J.J. (2020). Integrating artificial intelligence into the clinical practice of radiology: Challenges and recommendations. Eur. Radiol..

[23] Ahmad S., Khan S., AlAjmi M.F., Dutta A.K., Dang L.M., Joshi G.P., Moon H. (2022). Deep Learning Enabled Disease Diagnosis for Secure Internet of Medical Things. Comput. Mater. Contin..

[24] Ciresan D., Giusti A., Gambardella L., Schmidhuber J. (2012). Deep Neural Networks Segment Neuronal Membranes in Electron Microscopy Images. Advances in Neural Information Processing Systems.

[25] Ronneberger O., Fischer P., Brox T. (2015). U-net: Convolutional Networks for Biomedical Image Segmentation. Medical Image Computing and Computer-Assisted Intervention–MICCAI 2015.

[26] Haq A.U., Li J.P., Agbley B.L.Y., Khan A., Khan I., Uddin M.I., Khan S. (2022). IIMFCBM: Intelligent integrated model for feature extraction and classification of brain tumors using MRI clinical imaging data in IoT-healthcare. IEEE J. Biomed. Health Inform..

[27] Çiçek Ö., Abdulkadir A., Lienkamp S.S., Brox T., Ronneberger O. (2016). 3D U-Net: Learning Dense Volumetric Segmentation from Sparse Annotation. Medical Image Computing and Computer-Assisted Intervention—MICCAI 2016.

[28] Tong Q., Ning M., Si W., Liao X., Qin J. (2018). 3D Deeply-Supervised U-Net Based Whole Heart Segmentation. Statistical Atlases and Computational Models of the Heart. ACDC and MMWHS Challenges.

[29] Chen W., Liu B., Peng S., Sun J., Qiao X. (2019). S3D-UNet: Separable 3D U-Net for Brain Tumor Segmentation. Brainlesion: Glioma, Multiple Sclerosis, Stroke and Traumatic Brain Injuries.

[30] Kolarik M., Burget R., Uher V., Povoda L. Superresolution of MRI brain images using unbalanced 3D Dense-U-Net network. Proceedings of the 2019 42nd International Conference on Telecommunications and Signal Processing (TSP).

[31] Gamal A., Bedda K., Ashraf N., Ayman S., AbdAllah M., Rushdi M.A. Brain Tumor Segmentation using 3D U-Net with Hyperparameter Optimization. Proceedings of the 2021 3rd Novel Intelligent and Leading Emerging Sciences Conference (NILES).

[32] Yu W., Fang B., Liu Y., Gao M., Zheng S., Wang Y. Liver Vessels Segmentation Based on 3d Residual U-NET. Proceedings of the 2019 IEEE International Conference on Image Processing (ICIP).

[33] Owler J., Irving B., Ridgeway G., Wojciechowska M., McGonigle J., Brady S.M. (2020). Comparison of Multi-atlas Segmentation and U-Net Approaches for Automated 3D Liver Delineation in MRI. Medical Image Understanding and Analysis.

[34] González Sánchez J.C., Magnusson M., Sandborg M., Carlsson Tedgren Å., Malusek A. (2020). Segmentation of bones in medical dual-energy computed tomography volumes using the 3D U-Net. Phys. Medica.

[35] Yang Z. (2021). A Novel Brain Image Segmentation Method Using an Improved 3D U-Net Model. Sci. Program.

[36] He K., Zhang X., Ren S., Sun J. Deep residual learning for image recognition. Proceedings of the IEEE conference on computer vision and pattern Recognition (CVPR).

[37] Abdelaziz Ismael S.A., Mohammed A., Hefny H. (2020). An enhanced deep learning approach for brain cancer MRI images classification using residual networks. Artif. Intell. Med..

[38] Li H., Chen D., Nailon W.H., Davies M.E., Laurenson D. (2018). Improved Breast Mass Segmentation in Mammograms with Conditional Residual U-Net. Image Analysis for Moving Organ, Breast, and Thoracic Images.

[39] Wang G., Li W., Ourselin S., Vercauteren T. (2019). Automatic brain tumor segmentation using convolutional neural networks with test-time augmentation. Lect. Notes Comput. Sci. (Incl. Subser. Lect. Notes Artif. Intell. Lect. Notes Bioinform.).

[40] Zhang J., Lv X., Sun Q., Zhang Q., Wei X., Liu B. (2019). SDResU-Net: Separable and Dilated Residual U-Net for MRI Brain Tumor Segmentation. Curr. Med. Imaging.

[41] Saeed M.U., Ali G., Bin W., Almotiri S.H., AlGhamdi M.A., Nagra A.A., Masood K., Amin R. (2021). ul RMU-Net: A Novel Residual Mobile U-Net Model for Brain Tumor Segmentation from MR Images. Electronics.

[42] Abd-Ellah M.K., Khalaf A.A.M., Awad A.I., Hamed H.F.A. (2019). TPUAR-Net: Two Parallel U-Net with Asymmetric Residual-Based Deep Convolutional Neural Network for Brain Tumor Segmentation. Image Analysis and Recognition.

[43] Nguyen P.X., Lu Z., Huang W., Huang S., Katsuki A., Lin Z. Medical Image Segmentation with Stochastic Aggregated Loss in a Unified U-Net. Proceedings of the 2019 IEEE EMBS International Conference on Biomedical Health Informatics (BHI).

[44] Oktay O., Schlemper J., Folgoc L.L., Lee M., Heinrich M., Misawa K., Mori K., McDonagh S., Hammerla N.Y., Kainz B. (2018). Attention U-Net: Learning Where to Look for the Pancreas. arXiv.

[45] Vaswani A., Shazeer N., Parmar N., Uszkoreit J., Jones L., Gomez A.N., Kaiser Ł., Polosukhin I. (2017). Attention is All you Need. Advances in Neural Information Processing Systems.

[46] Schlemper J., Oktay O., Schaap M., Heinrich M., Kainz B., Glocker B., Rueckert D. (2019). Attention gated networks: Learning to leverage salient regions in medical images. Med. Image Anal..

[47] Fang Z., Chen Y., Nie D., Lin W., Shen D. (2019). RCA-U-Net: Residual Channel Attention U-Net for Fast Tissue Quantification in Magnetic Resonance Fingerprinting. Medical Image Computing and Computer Assisted Intervention—MICCAI 2019.

[48] Huang G., Liu Z., Van Der Maaten L., Weinberger K.Q. Densely connected convolutional networks. Proceedings of the 30th IEEE Conf. Comput. Vis. Pattern Recognition, CVPR 2017.

[49] Yang Z., Xu P., Yang Y., Bao B.K. (2021). A Densely Connected Network Based on U-Net for Medical Image Segmentation. ACM Trans. Multimed. Comput. Commun. Appl..

[50] Li S., Dong M., Du G., Mu X. (2019). Attention Dense-U-Net for Automatic Breast Mass Segmentation in Digital Mammogram. IEEE Access.

[51] Ji Z., Han X., Lin T., Wang W. A Dense-Gated U-Net for Brain Lesion Segmentation. Proceedings of the International Conference on Visual Communications and Image Processing (VCIP).

[52] Kolařík M., Burget R., Uher V., Dutta M.K. 3D Dense-U-Net for MRI Brain Tissue Segmentation. Proceedings of the 2018 41ST international conference on telecommunications and signal processing (TSP).

[53] Kolařík M., Burget R., Uher V., Říha K., Dutta M.K. (2019). Optimized high resolution 3D dense-U-Net network for brain and spine segmentation. Appl. Sci..

[54] Zhou Z., Rahman Siddiquee M.M., Tajbakhsh N., Liang J. (2018). Unet++: A nested u-net architecture for medical image segmentation. Deep Learning in Medical Image Analysis and Multimodal Learning for Clinical Decision Support.

[55] Hou A., Wu L., Sun H., Yang Q., Ji H., Cui B., Ji P. Brain Segmentation Based on UNet++ with Weighted Parameters and Convolutional Neural Network. Proceedings of the 2021 IEEE International Conference on Advances in Electrical Engineering and Computer Applications (AEECA).

[56] Micallef N., Seychell D., Bajada C.J. A Nested U-Net Approach for Brain Tumour Segmentation. Proceedings of the 2020 IEEE 20th Mediterranean Electrotechnical Conference (MELECON 2020)—Proceedings.

[57] Micallef N., Seychell D., Bajada C.J. (2021). Exploring the U-Net++ Model for Automatic Brain Tumor Segmentation. IEEE Access.

[58] Li C., Tan Y., Chen W., Luo X., He Y., Gao Y., Li F. (2020). ANU-Net: Attention-based nested U-Net to exploit full resolution features for medical image segmentation. Comput. Graph..

[59] Huang H., Lin L., Tong R., Hu H., Zhang Q., Iwamoto Y., Han X., Chen Y.W., Wu J. UNet 3+: A Full-Scale Connected UNet for Medical Image Segmentation. Proceedings of the ICASSP 2020 IEEE International Conference on Acoustics, Speech and Signal Processing (ICASSP).

[60] Goodfellow I., Pouget-Abadie J., Mirza M., Xu B., Warde-Farley D., Ozair S., Courville A., Bengio Y. (2014). Generative Adversarial Nets. Advances in Neural Information Processing Systems.

[61] Mirza M., Osindero S. (2014). Conditional Generative Adversarial Nets. arXiv.

[62] Chen X., Li Y., Yao L., Adeli E., Zhang Y. (2021). Generative Adversarial U-Net for Domain-free Medical Image Augmentation. arXiv.

[63] Li G., Zhang L., Hu S., Fu D., Liu M. Adversarial Network with Dual U-net Model and Multiresolution Loss Computation for Medical Images Registration. Proceedings of the 2019 12th International Congress on Image and Signal Processing, BioMedical Engineering and Informatics (CISP-BMEI).

[64] Yang G., Yu S., Dong H., Slabaugh G., Dragotti P.L., Ye X., Liu F., Arridge S., Keegan J., Guo Y. (2018). DAGAN: Deep De-Aliasing Generative Adversarial Networks for Fast Compressed Sensing MRI Reconstruction. IEEE Trans. Med. Imaging.

[65] Chen Y., Jakary A., Avadiappan S., Hess C.P., Lupo J.M. (2020). QSMGAN: Improved Quantitative Susceptibility Mapping using 3D Generative Adversarial Networks with increased receptive field. Neuroimage.

[66] Teki S.M., Varma M.K., Yadav A.K. (2019). Brain tumour segmentation using U-net based adversarial networks. Trait. Du Signal.

[67] Chen J., Lu Y., Yu Q., Luo X., Adeli E., Wang Y., Lu L., Yuille A.L., Zhou Y. (2021). TransUNet: Transformers Make Strong Encoders for Medical Image Segmentation. arXiv.

[68] Milletari F., Navab N., Ahmadi S.-A. V-Net: Fully Convolutional Neural Networks for Volumetric Medical Image Segmentation. Proceedings of the 2016 Fourth International Conference on 3D Vision (3DV).

[69] Alom M.Z., Yakopcic C., Hasan M., Taha T.M., Asari V.K. (2019). Recurrent residual U-Net for medical image segmentation. J. Med. Imaging.

[70] Fatemeh Z., Nicola S., Satheesh K., Eranga U. (2020). Ensemble U-net-based method for fully automated detection and segmentation of renal masses on computed tomography images. Med. Phys..

[71] Feng X., Wang C., Cheng S., Guo L. (2019). Automatic Liver and Tumor Segmentation of CT Based on Cascaded U-Net. Proceedings of 2018 Chinese Intelligent Systems Conference.

[72] Valanarasu J.M.J., Sindagi V.A., Hacihaliloglu I., Patel V.M. (2021). KiU-Net: Overcomplete Convolutional Architectures for Biomedical Image and Volumetric Segmentation. IEEE Trans. Med. Imaging.

[73] Zhang J., Lv X., Zhang H., Liu B. (2020). AResU-Net: Attention residual U-Net for brain tumor segmentation. Symmetry.

[74] Lecun Y., Bottou L., Bengio Y., Haffner P. (1998). Gradient-based learning applied to document recognition. Proc. IEEE.

[75] Dice L.R. (1945). Measures of the Amount of Ecologic Association Between Species. Ecology.

[76] Fidon L., Li W., Garcia-Peraza-Herrera L.C., Ekanayake J., Kitchen N., Ourselin S., Vercauteren T. (2018). Generalised Wasserstein Dice Score for Imbalanced Multi-class Segmentation Using Holistic Convolutional Networks. Brainlesion: Glioma, Multiple Sclerosis, Stroke and Traumatic Brain Injuries.

[77] Jaccard P. (1912). The distribution of the flora in the alpine zone.1. New Phytol..

[78] Abraham N., Khan N.M. A Novel Focal Tversky Loss Function With Improved Attention U-Net for Lesion Segmentation. Proceedings of the 2019 IEEE 16th International Symposium on Biomedical Imaging (ISBI 2019).

[79] Kervadec H., Bouchtiba J., Desrosiers C., Granger E., Dolz J., Ben Ayed I. Boundary loss for highly unbalanced segmentation. Proceedings of the 2nd International Conference on Medical Imaging with Deep Learning.

[80] Gerig G., Jomier M., Chakos M. (2001). Valmet: A New Validation Tool for Assessing and Improving 3D Object Segmentation. Medical Image Computing and Computer-Assisted Intervention—MICCAI 2001.

[81] Nai Y.H., Teo B.W., Tan N.L., O’Doherty S., Stephenson M.C., Thian Y.L., Chiong E., Reilhac A. (2021). Comparison of metrics for the evaluation of medical segmentations using prostate MRI dataset. Comput. Biol. Med..

[82] Taha A.A., Hanbury A. (2015). Metrics for evaluating 3D medical image segmentation: Analysis, selection, and tool. BMC Med. Imaging.

[83] Menze B.H., Jakab A., Bauer S., Kalpathy-Cramer J., Farahani K., Kirby J., Burren Y., Porz N., Slotboom J., Wiest R. (2014). The multimodal brain tumor image segmentation benchmark (BRATS). IEEE Trans. Med. Imaging.

[84] Isensee F., Jäger P.F., Full P.M., Vollmuth P., Maier-Hein K.H. (2020). nnU-Net for brain tumor segmentation. International MICCAI Brainlesion Workshop.

[85] Sahayam S., Nenavath R., Jayaraman U., Prakash S. (2022). Brain tumor segmentation using a hybrid multi resolution U-Net with residual dual attention and deep supervision on MR images. Biomed. Signal Process. Control.

[86] Maji D., Sigedar P., Singh M. (2022). Attention Res-UNet with Guided Decoder for semantic segmentation of brain tumors. Biomed. Signal Process. Control.

[87] Ellis D.G., Aizenberg M.R. (2020). Trialing u-Net Training Modifications for Segmenting Gliomas Using Open Source Deep Learning Framework. International MICCAI Brainlesion Workshop.

[88] Qamar S., Ahmad P., Shen L. (2020). Hi-net: Hyperdense Inception 3d Unet for Brain Tumor Segmentation. International MICCAI Brainlesion Workshop.

[89] Cirillo M.D., Abramian D., Eklund A. (2020). Vox2Vox: 3D-GAN for Brain Tumour Segmentation. International MICCAI Brainlesion Workshop.

[90] Ahmad P., Qamar S., Shen L., Saeed A. (2020). Context aware 3D UNet for Brain Tumor Segmentation. International MICCAI Brainlesion Workshop.

[91] Agarwala S., Sharma S., Uma Shankar B. A-UNet: Attention 3D UNet architecture for multiclass segmentation of Brain Tumor. Proceedings of the 2022 IEEE Region 10 Symposium (TENSYMP).

[92] Raza R., Bajwa U.I., Mehmood Y., Anwar M.W., Jamal M.H. (2023). dResU-Net: 3D deep residual U-Net based brain tumor segmentation from multimodal MRI. Biomed. Signal Process. Control.

[93] AboElenein N.M., Songhao P., Afifi A. (2022). IRDNU-Net: Inception residual dense nested u-net for brain tumor segmentation. Multimed. Tools Appl..

[94] Ghaffari M., Sowmya A., Oliver R. (2021). Automated Brain Tumour Segmentation Using Cascaded 3D Densely-Connected U-Net BT—Brainlesion: Glioma, Multiple Sclerosis, Stroke and Traumatic Brain Injuries.

[95] Wang W., Chen C., Ding M., Yu H., Zha S., Li J. Transbts: Multimodal brain tumor segmentation using transformer. Proceedings of the International Conference on Medical Image Computing and Computer-Assisted Intervention.

[96] Henry T., Carré A., Lerousseau M., Estienne T., Robert C., Paragios N., Deutsch E. (2020). Brain Tumor Segmentation with Self-Ensembled, Deeply-Supervised 3D U-Net Neural Networks: A BraTS 2020 Challenge Solution. International MICCAI Brainlesion Workshop.

[97] Zuo Q., Chen S., Wang Z. (2021). R2AU-Net: Attention recurrent residual convolutional neural network for multimodal medical image segmentation. Secur. Commun. Netw..

[98] Chen L.C., Papandreou G., Kokkinos I., Murphy K., Yuille A.L. (2018). DeepLab: Semantic Image Segmentation with Deep Convolutional Nets, Atrous Convolution, and Fully Connected CRFs. IEEE Trans. Pattern Anal. Mach. Intell..

[99] Peiris H., Chen Z., Egan G., Harandi M. (2022). Reciprocal adversarial learning for brain tumor segmentation: A solution to BraTS challenge 2021 segmentation task. arXiv.

[100] Hussain Z., Gimenez F., Yi D., Rubin D. (2017). Differential Data Augmentation Techniques for Medical Imaging Classification Tasks. AMIA Annu. Symp. Proc. AMIA Symp..

[101] Goodfellow I., Bengio Y., Courville A. (2016). Deep Learning?. Nature.

[102] Yi X., Walia E., Babyn P. (2019). Generative adversarial network in medical imaging: A review. Med. Image Anal..

[103] Papernot N., McDaniel P., Goodfellow I., Jha S., Celik Z.B., Swami A. Practical Black-Box Attacks against Machine Learning. Proceedings of the 2017 ACM on Asia Conference on Computer and Communications Security.

